# The intervention of Brain Gym in the mathematical abilities of high-school students: A pilot study

**DOI:** 10.3389/fpsyg.2022.1045567

**Published:** 2023-01-15

**Authors:** Carlos Ramos-Galarza, Cristina Aymacaña-Villacreses, Jorge Cruz-Cárdenas

**Affiliations:** ^1^Facultad de Psicología, Pontificia Universidad Católica del Ecuador, Quito, Ecuador; ^2^Centro de Investigación MIST, Carrera de Psicología, Universidad Tecnológica Indoamérica, Quito, Ecuador; ^3^Centro de Investigación ESTec, Universidad Tecnológica Indoamérica, Quito, Ecuador

**Keywords:** Brain Gym, Mathematics, problem-solving, cognitive skills, fractions

## Abstract

**Introduction:**

The learning process of Mathematics is a challenge in Latin America; therefore, it is of vital importance to conduct actions that improve the performance in this science.

**Methods:**

This article is reporting on quasi-experimental research, where, through the use of Brain Gym, the objective is to improve: the definition of rational numbers, problem-solving ability, mathematical order relationships, and equivalent fractions. We worked with 67 students between 12 and 14 years old, organized into an experimental group (*n* = 35) and a control group (no participation group; *n* = 32).

**Results:**

We made pre and post-test measurements and found that the control group students improved in their problem-solving ability *F*(1,65) = 8.76, *p* = 0.04, *η*^2^ = 0.12 and equivalent fractions *F*(1,65) = 4.54, *p* = 0.03, *η*^2^ = 0.06.

**Discussion:**

In conclusion, the importance of applying innovative processes to improve the teaching and learning of Mathematics can be affirmed. It is important to note that both the control and experimental groups improved their learning, however, the experimental group did so to a greater extent than the students in the control group, who received a traditional educational process, and they did learn, but not at the level of the experimental group.

## Introduction

Mathematics is a science that strengthens the progress and scientific advancement of society. Its study has become necessary and essential for the improvement of people’s life quality, organizations, and the economic development of countries for centuries (Chavarría-Arroyo and Albanese, 2021). In the educational context, it is important to relate it to social and cultural life. In addition, it contributes to the curiosity and creativity of students to solve environmental problems with critical, reflective, analytical, systematic, numerical, and logical thinking (Ministry of Education [MINEDUC], 2019).

As part of the knowledge of Mathematics, the understanding of the definition and elements of rational numbers helps to strengthen the thought units that give meaning to those situations where there is a division of the unit of the numerator and denominator. The order relationship strengthens the analysis of the numerical value to establish a comparison between greater, lesser, or equal several rational numbers. By recognizing and constructing equivalent fractions with the ability to calculate through amplification and simplification, it is possible to obtain another fraction with the same value (Ministry of Education [MINEDUC], 2019).

Problem-solving develops critical, reflective, and metacognitive thinking. This mathematical ability starts from reading comprehension, planning of the resolution process, the execution of the planning, in which the mathematical calculations are executed, and finishes with the verification of the answer. This mathematical knowledge is essential to develop life learning skills (Torregrosa et al., 2020).

In spite of the great importance of learning Mathematics, its learning and teaching process has difficulties that generate a negative impact on cognitive skills, critical thinking, and calculation, and at the same time, make students avoid wanting to solve difficult mathematical problems (Adiastuty, 2021). In recent decades, there has been research that has detected that the educational model fails to achieve the learning skills of Mathematics. In addition, it is labeled as a difficult subject that is scary and boring. As result, it has become a concern for teachers, educators, and researchers worldwide (García, 2013; Cai and Mok, 2017).

According to results of evaluations in Mathematics worldwide, it has been reported that countries such as Singapore, China, and the United States lead the learning of this science (González-Mayorga et al., 2022). In Latin America, where this research is done, it has been found that countries such as Uruguay and Chile lead the performance in Mathematics, while, ranking lower, we have countries such as Ecuador, with a lower performance in calculation skills (Instituto Nacional de Evaluación Educativa [INEVAL], 2018; Organization for Economic Co-operation and Development, 2019).

Similarly, previous studies reveal that the student’s lack of cognitive skills in problem-solving, development of critical thinking, and calculation exercises can cause professional failure, which motivates a percentage of teachers to be interested in developing innovative didactic learning strategies that are significant and useful. And at the same time, provide solutions to cognitive difficulties, especially in the area of Mathematics. They also improve the repercussions that could be generated in society (Mulyanto, 2018; González and Rodríguez, 2022).

Within these innovative activities to contribute to the solution of the problem of the teaching and learning of Mathematics, the application of Brain Gym arises as a new experience that, through the brain-movement-body connection, accelerates the learning of it. Significantly, supporting the lack of attention, memory, comprehension, and organization problems. In addition, research shows that Brain Gym reduces the stress that can be generated by learning the different contents of Mathematics slowly or difficultly. Therefore providing an active strategy that strengthens the student’s cognitive capacity (Marpaung, 2017).

Brain Gym is a set of movements whose objective is to connect the body and the mind, stimulate the use of the cerebral hemispheres through physical and mental strategies, and also improve and strengthen cognitive functions to learn. It is part of Kinesiology and it’s the result of the research of Applied Neuroscience that studies the movements of the body and its relationship with brain function. It also allows stimulating and activating the cognitive process of an individual (Hoffman, 2009; Spaulding et al., 2010; Ferré, 2016; Marpaung, 2017; Ministry of Education [MINEDUC], 2020).

The Brain Gym program is applied through the PACE process, whose acronym stands for positive, active, clear, and energetic. It corresponds to the sequence of the four essential qualities that prepare a student for integrated learning in the brain. The chosen exercises are used in a basic way to maintain a balance in daily life, respecting rhythm and times. The qualities of being energetic, clear, active, and positive are related to the three types of movements that come from the central line, dividing the body into two equal parts: left–right, anterior–posterior, and up-down. These movements are based on the dimensions that become the pillar base to the understanding of how learning works, while applying the exercises of Brain Gym (Brain Gym International, 2018).

In the Brain Gym method, it is assumed that the brain can be trained and become more efficient through motor exercises, practically being like a muscle that needs to be exercised to perform better (Groose, 2013). With the exercises of this method, a better connection between the left and right hemispheres is promoted, thus achieving a neuronal unlocking and favoring the learning process and neuronal synapsis (Jalilinasab et al., 2021).

Another element that favors at the cerebral level has to do with the activation of tone and wakefulness that human beings need to carry out a cognitive activity. In this case, the exercises increase the alert state of the subject, which will have a positive impact on functions such as attention, since by finding a better cortical tone, the subject will be able to concentrate more and improve their information processing (Ramos-Galarza et al., 2021).

The key to Brain Gym brain stimulation is based on how brain connections are activated with motor exercises and through the visual and auditory stimulation proposed by this method. What it would generate in the student’s mind is an optimal balanced state for their learning to take place, as is the case of this research, in the mathematics area (Spaulding et al., 2010).

The use of Brain Gym helps the human being in various areas in which he develops, such as education, sociability and health, among others. Investigations such as that of Arbianingsih et al. (2021) report the reduction of anxiety in hospitalized children of preschool and school-age, after applying Brain Gym exercises. According to Anggraini and Dewi (2022), concentration increases significantly, improving the teaching and learning process with the use of Brain Gym. At the level of academic performance of university students, according to Ramírez et al. (2021), the influence of Brain Gym exercises applied as a didactic strategy in Physical Education, reported that this type of intervention increases the performance in Mathematics and Communication. Other studies have reported the positive effects of applying Brain Gym in various populations to treat mental health problems and improve life quality (Surita et al., 2021).

Various studies have highlighted the benefit of the Brain Gym method, for example, it has been reported that this type of stimulation benefits motor and social skills (Jalilinasab et al., 2021), quality of life (Andi et al., 2019), skills (Kulkarni and Khandale, 2019), improves health balance in patients with neuropathies (Panse et al., 2018), improves concentration for online learning (Pratiwi and Pratama, 2020; Anggraini and Dewi, 2022), and reduction of fatigue and muscle pain (Ismayenti et al., 2021) among other research that indicates the positive contribution of this method.

In Latin America, little research has been carried out regarding the performance of students in Mathematics with Brain Gym. This study makes an important contribution to the line of research that seeks to improve mathematics performance in the basic educational context, since there is a very low level of performance in this subject in the region. This study is novel since it seeks to verify the effectiveness of the Brain Gym technique through a study with a quasi-experimental scope, which will allow contrasting the benefit of its application in favor of mathematical skills. For this reason, this study seeks to address four key elements of learning Mathematics: (a) the definition and elements of rational numbers, (b) problem-solving, (c) order relation and (d) equivalent fractions, which with the application of the Brain Gym program, through systematical exercises, the neuronal connections of the cerebral hemispheres are stimulated, significantly strengthening and improving the learning process.

## Investigation hypotheses

*H1*: The group of students that will receive the intervention procedure in Brain Gym will present better results in definition and elements of rational numbers when compared in the post-test measurement with the students of the control group.

*H2*: The group of students from the experimental group that will receive the Brain Gym intervention will have a higher level of problem-solving when compared in the post-test assessment with the students from the control group.

*H3*: The students of the experimental group will present higher levels concerning order when compared in the post-test with the students of the control group.

*H4*: The students of the group that receives the intervention with Brain Gym, will present better levels in equivalent fractions when compared in the post-test with the students of the control group.

## Materials and methods

### Research design

In this research, a quasi-experimental research design was used. Participants were divided into two groups, one that received a Brain Gym intervention and one that was a comparison control group.

### Sample

Two groups of the same level of education were randomly chosen with a total of 67 students, 35 (52%) females, and 32 (48%) males. The age of the participants was from 12 to 14 years old. The experimental group consisted of 35 students, 15 males, and 20 females. The control group was made up of 32 students, 17 males, and 15 females. Both the experimental and control groups had the same sociodemographic, economic, educational, and social characteristics. The characteristics of both groups matched.

In the G*Power program (Heinrich Heine Universität Düsseldorf, 2022) based on the sample size of 65 participants and with the parameters of median effect size 0.15, error probability of .05, 2 groups and 4 measurements made, it was found that, the study with the sample that worked has an adequate statistical power of *1-β* = 0.82.

### Measurements

The instruments used to support the research data were conducted through a test that was made up of 12 components that measured the knowledge of the four topics of rational numbers, which were then checked by experts in Mathematics. These were in the Forms app, to be applied online to the experimental group and the control group before and after the intervention.

### Intervention protocol

The intervention consisted of a program made up of Brain Gym exercises aligned to improve the learning of Mathematics. Then, they were organized based on the PACE process and incorporated into the pedagogical day during the sessions that the intervention program lasted. In the intervention with the experimental group, a series of Brain Gym exercises were carried out that allowed the student to generate a better brain function to perform in mathematics.

The exercises performed were: (a) crossed crawl, consisted of energetically touching the left knee with the right elbow and in a contrary way. This exercise allowed the activation of the brain to improve visual, auditory, kinesiological and tactile abilities. (b) The owl, puts its hand on the shoulder of the opposite side, squeezing it firmly and turns its head towards that side. The student must take a deep breath and exhale by turning the head towards the opposite shoulder. The same is done with the other side (Dennison, 1992; Kulkarni and Khandale, 2019).

The next exercise in the intervention program was: (c) double scribbling, in which the student must draw with both hands at the same time, in, out, up, and down. This exercise benefits writing and fine motor skills. (d) Buttons of the brain, one hand is placed on the navel and with the other hand imaginary buttons must be drawn at the junction of the clavicle with the sternum, making circular movements in a clockwise direction. These exercises stimulate the eyesight and improve bilateral coordination (Dennison, 1992; Murtadho et al., 2019).

Subsequently, the exercises were applied: (e) energetic yawning, where the fingertips are placed on the cheeks, a yawn is simulated and pressure is applied with the fingers. This exercise stimulates verbal expression and communication. The brain is oxygenated, relaxes tension in the facial area and improves visual perception. (f) Eight lazy or lying down, a large eight must be drawn horizontally or with pencil and paper. Begin to draw the center and continue to the left, return to the center and finish on the right side. Finally, (g) thinking hat, you should put your hands on your ears and try to follow the shape of the ear from the ear canal out. These exercises help listening skills, improve attention, verbal fluency and balance (Dennison, 1992; Pratiwi and Pratama, 2020). Table 1 describes the applied protocol.

**Table 1:** Brain Gym intervention protocol.

Sessions	Objectives	Experimental group activities	Control group activities	Means	Time (min)
First	Diagnose the level of knowledge by applying an evaluation on rational numbers	Apply initial evaluation on rational numbers	Apply initial evaluation on rational numbers	Test in forms	40
Second	Recognize the set of Q rational numbers and identify their elements	Using the Brain Gym program, the set of rational numbers and their elements are presented	Presentation of the set of rational numbers and their elements passively and traditionally	Internet power point presentation	40
Third	Establish order relationships using the number line	Applying the Brain Gym program, fractions are located on the number line	Location of fractions on the number line passively and traditionally	Internet number line	40
Fourth	Solve rational numbers through problem-solving	Using the Brain Gym program, the problem-solving method is applied	Application of the method for solving problems passively and traditionally	Internet white board markers	40
Fifth	Establish equivalent fractions from a fraction	Applying the Brain Gym program, equivalent fractions are done	Construction of equivalent fractions through amplification passively and traditionally	Internet PhET tool	40
Sixth	Form irreducible fractions from simplification	Fractions are simplified using the Brain Gym program	Simplification of fractions passively and traditionally	Internet white board sheets	40
Seventh	Establish order relationships in a set of rational numbers	Using the Brain Gym program, equivalent fractions are done	Construction of equivalent fractions passively and traditionally	Internet video	40
Eighth	Evaluate the level of knowledge by applying an evaluation on rational numbers	Apply final evaluation on rational numbers	Apply final evaluation on rational numbers	Test in forms	40

**Figure 1:**
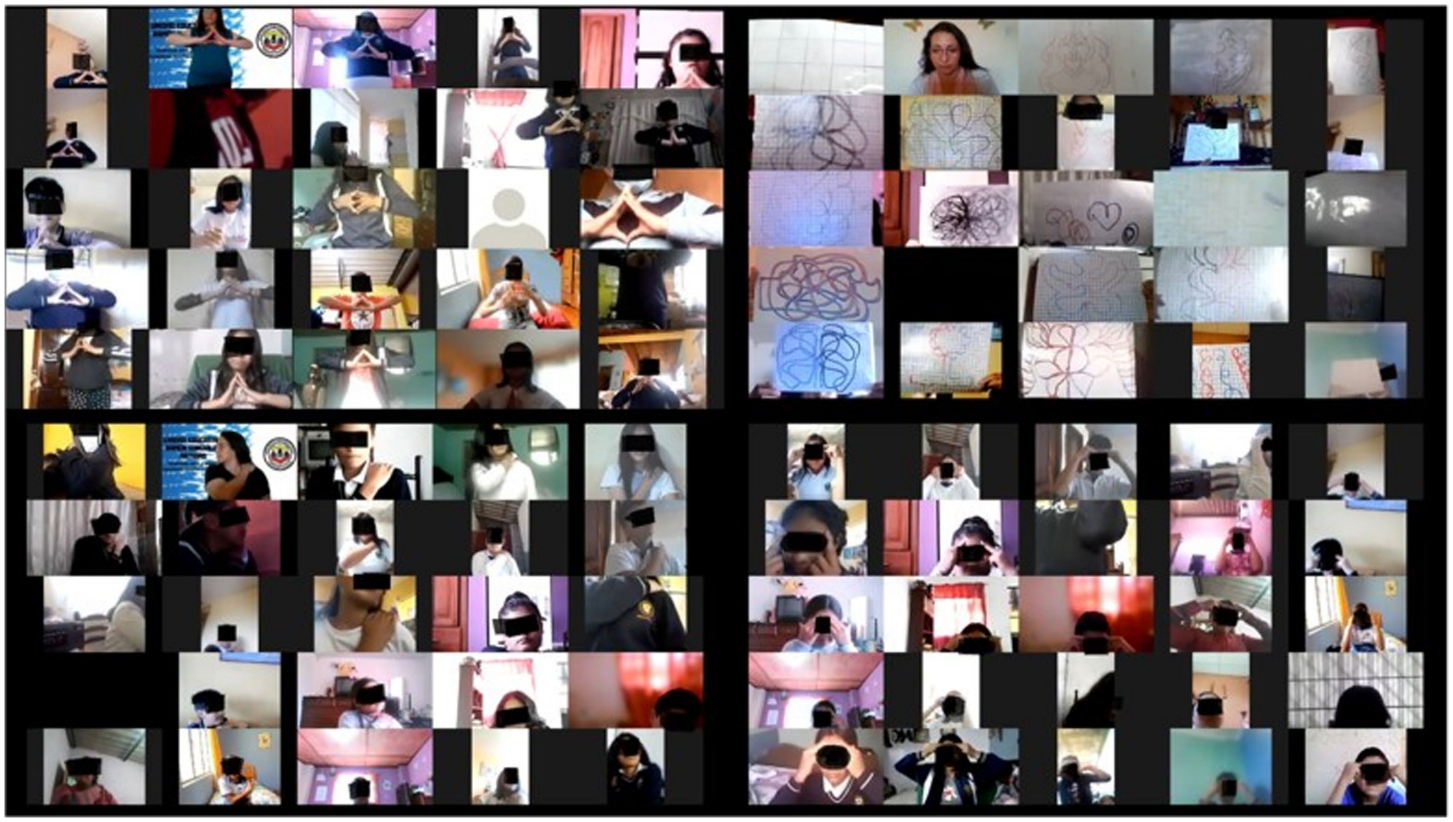
Execution of Brain Gym exercises.

The intervention lasted 8 weeks within the mathematics class of two courses. This process was carried out during the online class process, where the execution of the exercises was explained to the students of the control group. In each of the sessions, the students were supervised by their parents and the teacher on the correct execution of the Brain Gym exercises performed. In Figure 1 you can see images of the intervention performed.

### Procedure

The investigation began with the approval from the authority of the institution and the ethics committee of the Indoamerica University of Ecuador. Two groups of the same educational level, an experimental group, and a control group were randomly chosen. The intervention program began with the application of a pre-test to the two groups, who had knowledge of the four key elements of Mathematics. Then, the Brain Gym program was applied for eight sessions. Once the intervention program finished, they were evaluated with a post-test with the same knowledge that was measured at the beginning.

### Statistical analysis

The analysis began with descriptive statistics to characterize the quantitative results of the participants. After that, a 2×2 ANOVA was done. The control and experimental groups were considered as two levels of the factors between them. In the intra-group factor, the two pre and post-test levels were considered. The comparisons with the Bonferroni statistic were used as a model adjustment and the level of significance was valued at *p* < 0.05. All analyzes were performed in the SPSS version 28 program. These analyses were carried out in the statistical package SPSS v. 25.

### The study scenario

This study was conducted in the capital of Ecuador, the city of Quito. Ecuador is a middle-income developing country located in South America. Ecuador has an approximate population of 17 million inhabitants, while the city of Quito has, in its metropolitan area, a population close to 3 million inhabitants. Ecuador’s economic system is based on the private property model, and its official currency is the U.S. dollar (National Institute of Statistics and Censuses (INEC), Ecuador, 2013; National Institute of Educational Evaluation (INEE), Ecuador, 2019). Thus, the following presented results could be useful not solely in the context in which this research took place but also in other contexts that share similar described characteristics.

## Results

In the first place, the reliability of all the measurements carried out was calculated, for which the Cronbach’s Alpha statistic was used. Acceptable levels of internal consistency were found in this analysis. In addition, it was found that the items that build each of the variables were significantly correlated and contributed in favor of the construction of each scale. The reliability results of each variable are: definition and elements of post-test rational numbers α = 0.71, problem-solving pre-test α = 0.75, Post-test problem-solving α = 0.78, pre-test Order Relation α = 0.70, post-test Order Relation α = 0.70, pre-test equivalent fractions α = 0.76 and post-test equivalent fractions α = 0.70. Table 2 shows the descriptive results obtained by the control and intervention groups in the 4 variables assessed.

**Table 2:** Descriptive statistics.

	Group	Mean	SD
Definition and elements of rational numbers pre-test	Experimental group	7,6,000	1.94331
	Control group	7.5313	1.56544
Definition and elements of post-test rational numbers	Experimental group	8,5,714	0.88403
	Control group	8,0938	1.39952
Problem-solving pre-test	Experimental group	6.1143	1.07844
	Control group	6.0625	1.21649
Post-test problem-solving	Experimental group	7,4,000	1.49902
	Control group	5.9375	1.41279
Pre-test order relation	Experimental group	6,4,000	1.45925
	Control group	6.0625	1.21649
Post-test order relation	Experimental group	7.7143	1.34101
	Control group	6.8125	1.40132
Pre-test equivalent fractions	Experimental group	6.9143	1.50238
	Control group	6.7188	1.61114
Post-test equivalent fractions	Experimental group	8,4,286	0.88403
	Control group	7.3125	1.76777

**Figure 2:**
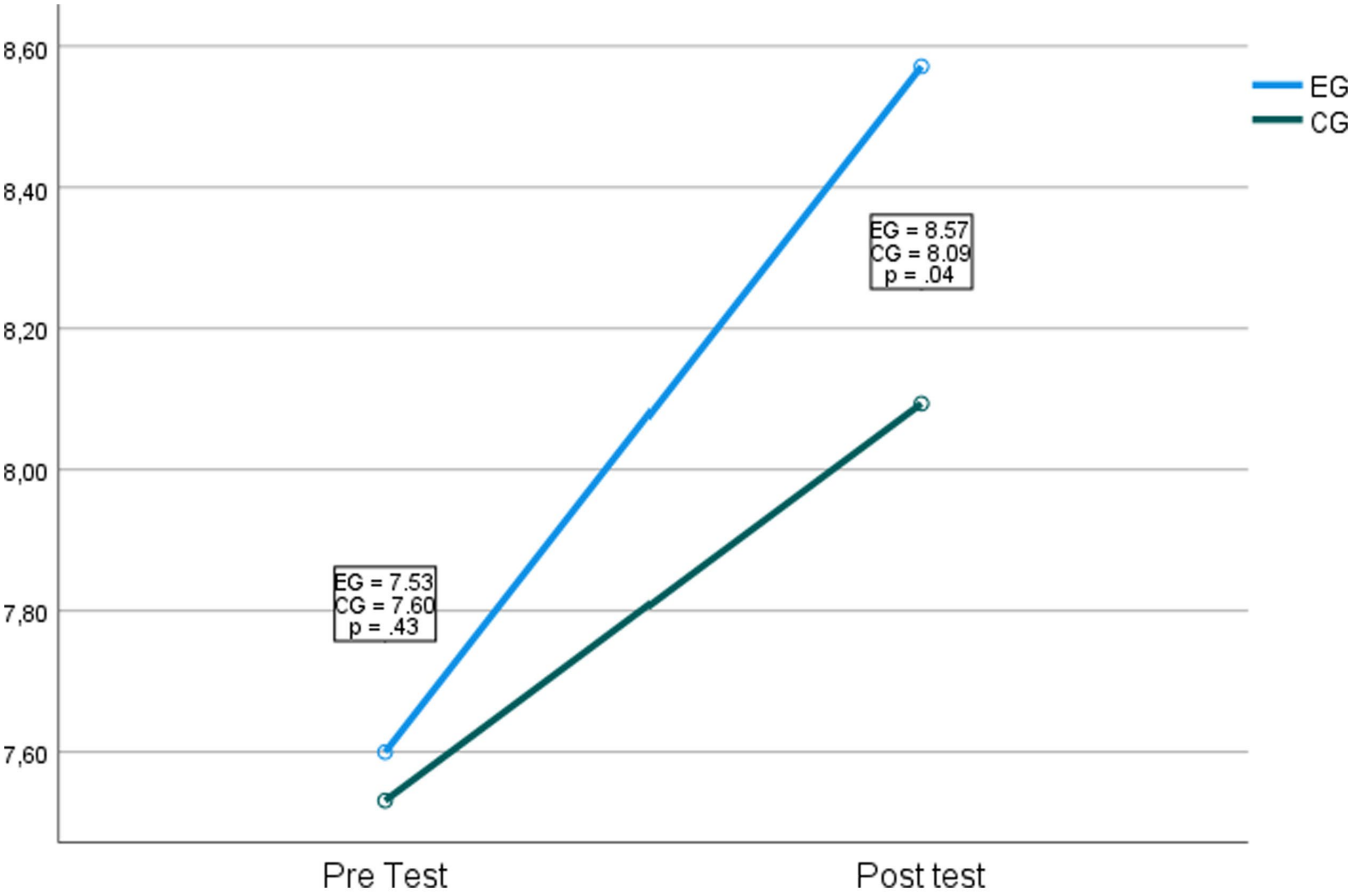
Comparison of the control and experimental groups in definition and elements.

Regarding the first hypothesis: the group of students belonging to the experimental group present higher levels in definition and elements when compared to the students of the control group. In the ANOVA, it was found that there are no differences in the interaction between the intervention and the pre and post-test measurement time *F*_(1,65)_ = 0.83, *p* = 0.36, *η*^2^ = 0.013. Significant effects of the technological intervention were found on the definition and elements of rational number items *F*_(1,65)_ = 3014.05, *p* = <0.001, *η*^2^ = 0.98. Figure 2 shows the comparison.

**Figure 3:**
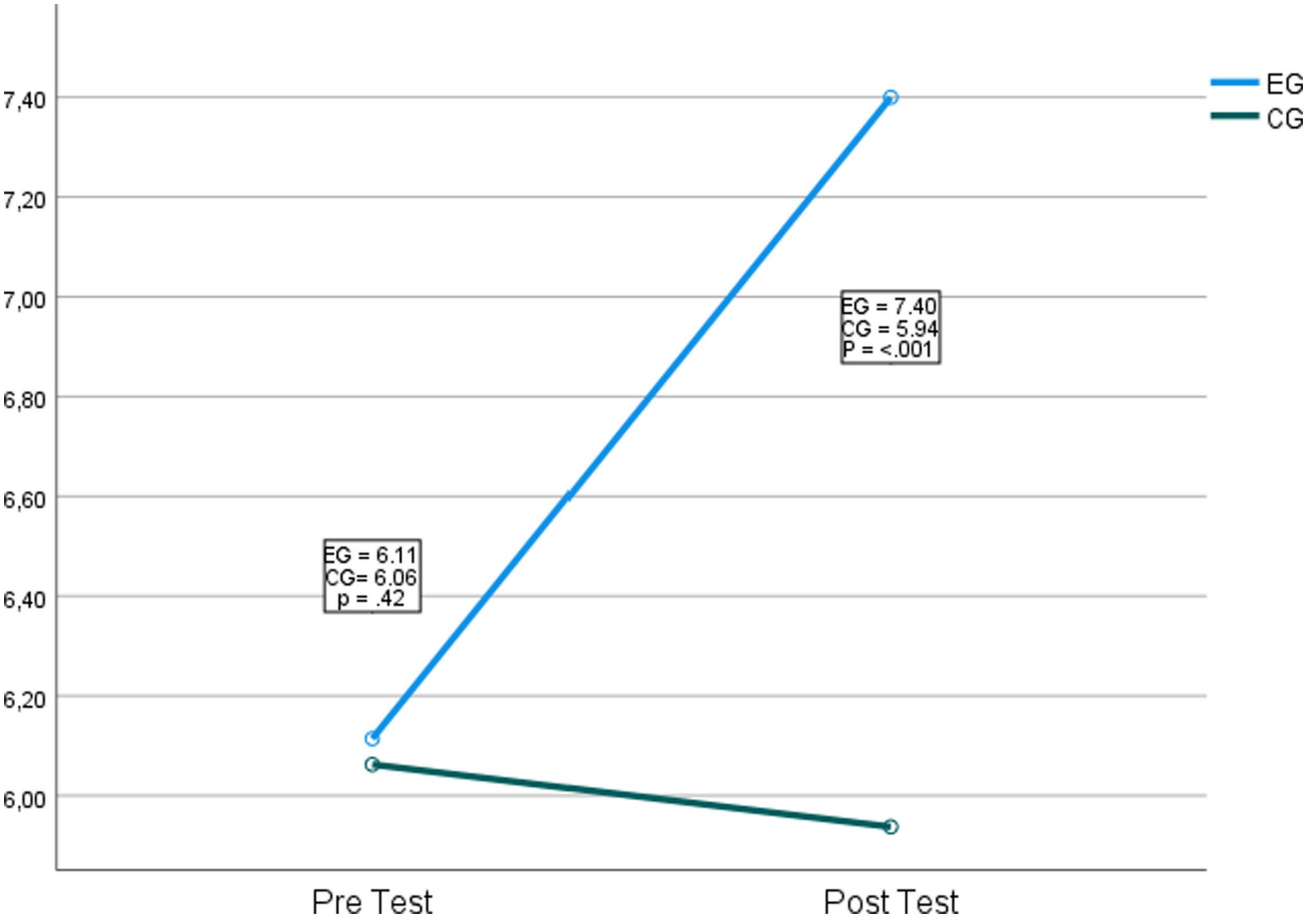
Comparison of the control and experimental groups in problem-solving.

Regarding the second hypothesis, which indicates that the participants of the experimental group will have higher levels of problem-solving, a significant interaction was found with the best levels of problem-solving in favor of the experimental group *F*_(1,65)_ = 8.76, *p* = 0.04, *η*^2^ = 0.12. In main effects, it was found that different approaches used with the experimental group positively influence the problem-solving ability of the students *F*_(1,65)_ = 3523.57, *p* = <0.001, *η*^2^ = 0.98. Figure 3 shows the graph of the comparison.

**Figure 4:**
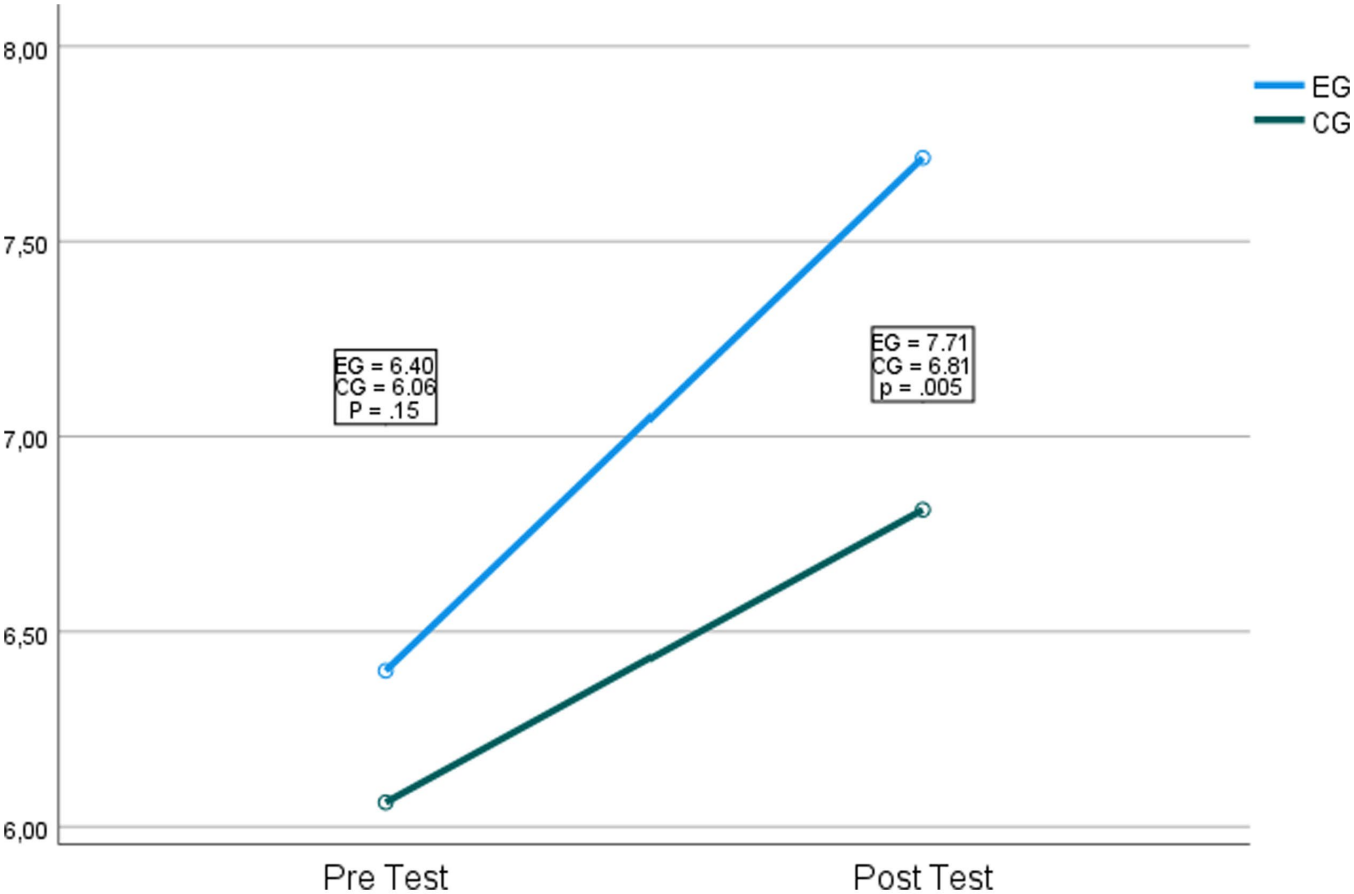
Comparison of the control and experimental groups concerning order.

Concerning the third hypothesis that stated that the students who received the different approaches would improve in relation to the order relation, it was found that there is no interaction between the intervention and the dependent variable *F*_(1,65)_ = 1.44, *p* = 0.23, *η*^2^ = 0.22. Concerning the main effects of the intervention on the order relation, statistically significant results were found *F*_(1,65)_ = 3283.56, *p* = <0.001, *η*^2^ = 0.96. Figure 4 shows the comparison.

**Figure 5:**
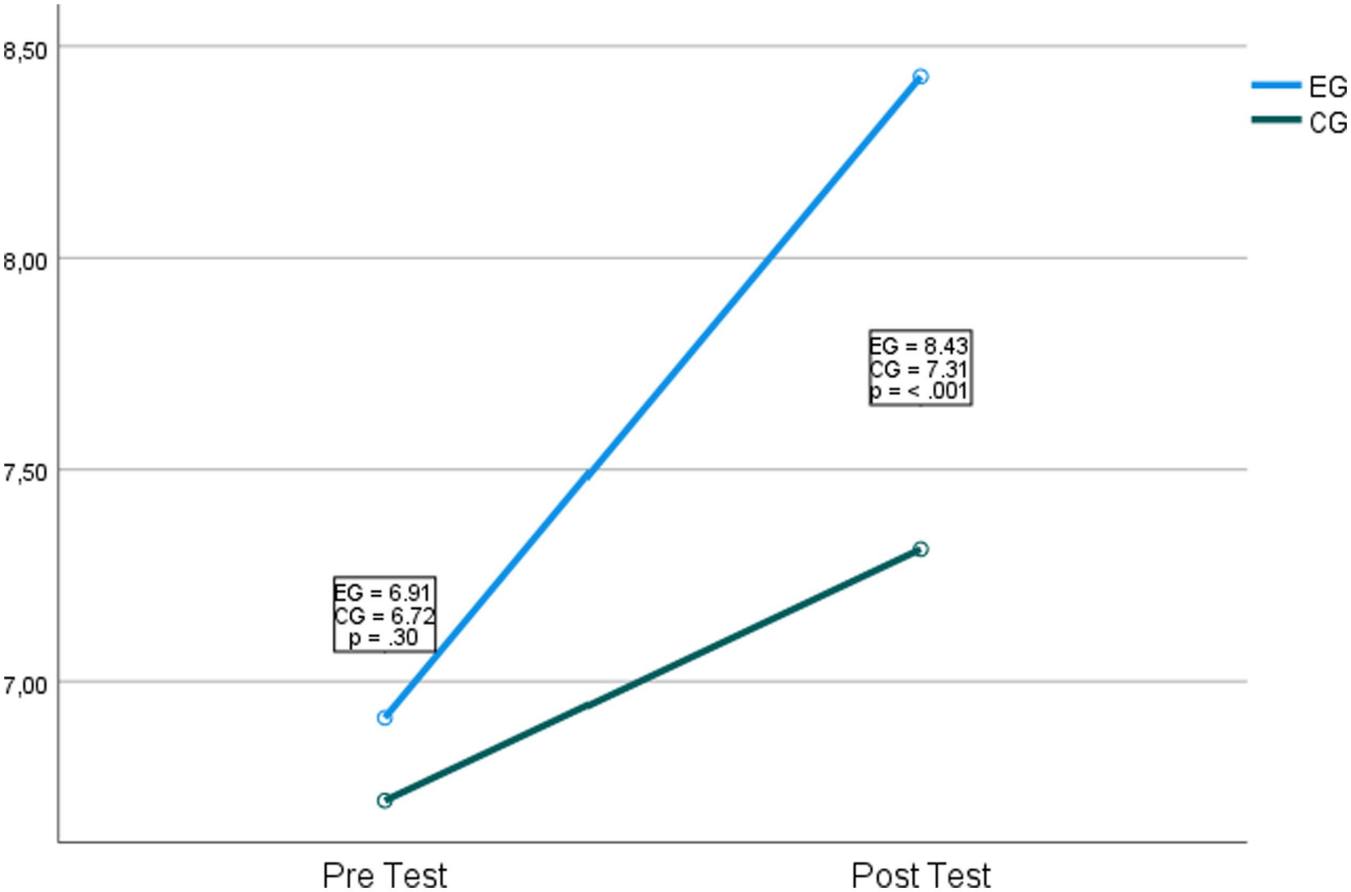
Comparison of the control and experimental groups in fractions.

Regarding the fourth hypothesis, which states that the students in the experimental group have better results in fractions when compared to the control group, a statistically significant interaction was found between the approaches and the best results in fractions with the students in the experimental group *F*_(1,65)_ = 4.54, *p* = 0.03, *η*^2^ = 0.06. Regarding the main effects, statistically significant results were found with the different approaches and performance in fractions *F*_(1,65)_ = 2612.56, *p* = <0.001, *η*^2^ = 0.97. Figure 5 shows the comparison.

**Figure 6:**
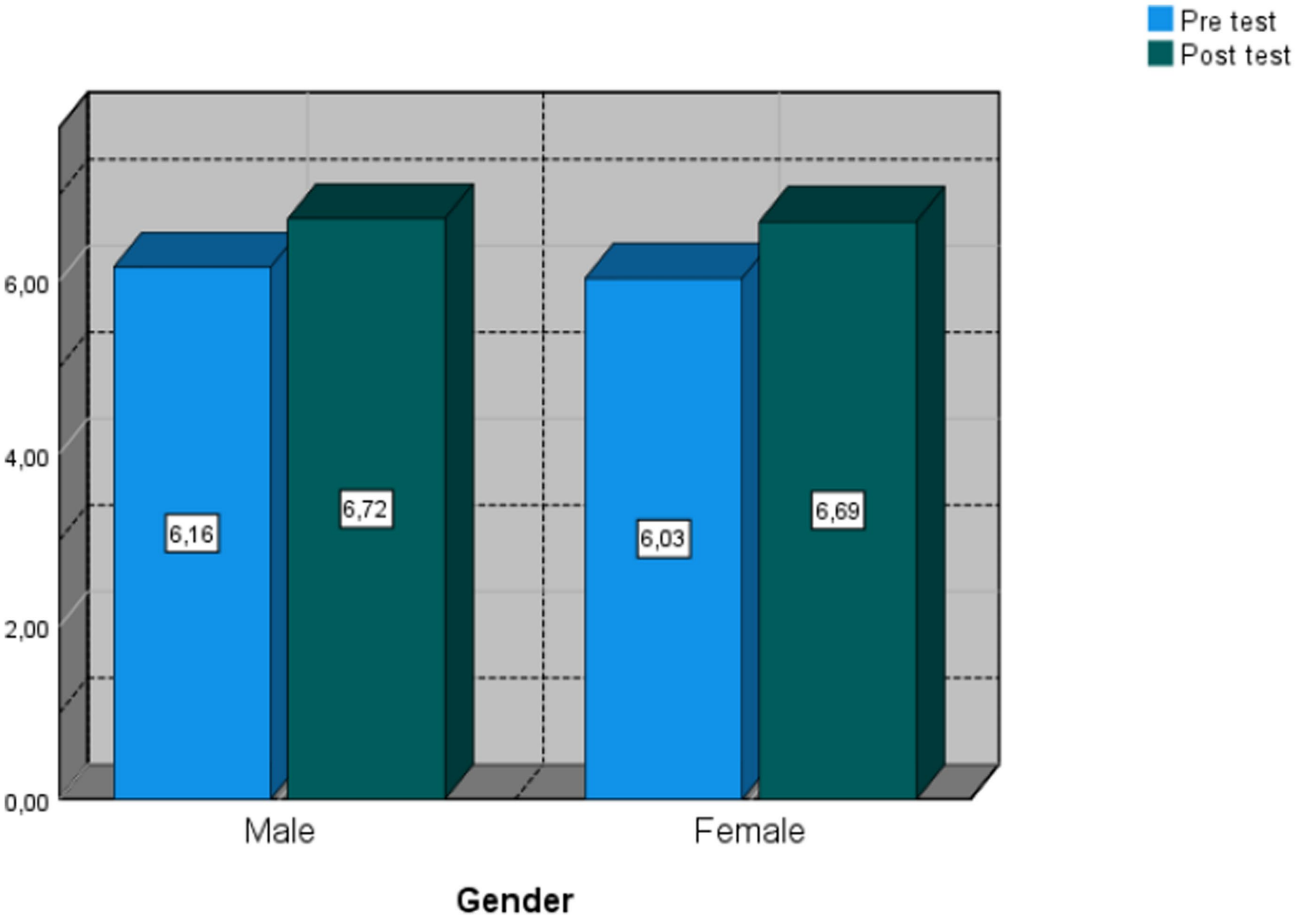
Scores in solving mathematical problems found in the pre- and post-test measurements.

An analysis was carried out considering the influence of gender on the measurements made. It was found that there is no statistically significant association between gender and definition and elements pre-test (*x^2^* =, *p* = 21) and post-test (*x^2^* = 5.75, *p* = 0.33), order relation pre-test (*x^2^* = 4.39, *p* = 0.62) and post-test (*x^2^* = 7.24, *p* = 0.20), equivalent fractions pre-test (*x^2^* = 8.19, *p* = 0.22) and post-test (*x^2^* = 6.96, *p* = 0.32). When relating gender to problem solving, a statistically significant association was found with problem solving pre-test (*x^2^* = 12.09, *p* = 0.03) and post-test (*x^2^* = 12.81, *p* = 0.04). Figure 6 shows the problem solving score according to the gender of the participants.

## Discussion

In the present investigation, the impact of Brain Gym on the learning process of Mathematics was demonstrated through the application of a quasi-experimental investigation measuring the understanding, definition, and knowledge of the elements of rational numbers, their order relation, equivalent fractions, and problem-solving. In the intervention process, an experimental group received different approaches using Brain Gym methods. On the other hand, with the control group, a traditional and passive methodology was used.

It is important to consider that Brain Gym which is an active strategy that allows the stimulation of the neuronal connections through the interaction of body–brain movement, allowed to increase neuroplasticity which at the same time significantly improved and benefited the learning process of the contents of rational numbers.

The results obtained in the research provide empirical evidence in favor of the hypotheses raised in this research regarding learning Mathematics better, specifically, the knowledge of rational numbers. When comparing the results between the experimental group and the control group, a significant difference is shown through the pre-test, the application of the Brain Gym program, and the post-test. In addition, the two groups improved their learning, nevertheless, in the control group, it does not significantly increase the way it does in the experimental group.

The results of this research are consistent with other previously conducted studies where the benefits of Brain Gym in the learning process of Mathematics are demonstrated. According to Colina et al. (2021) and Romero et al. (2021), with the Brain Gym program, the improvement of the learning process in students is obtained with positive, tangible, and measurable results, causing progress in all areas of knowledge.

In addition, the results found in this research would be explained by the benefit of the Brain Gym in cognitive skills such as attention, perception and memory of the human being, which could have influenced the better performance of the experimental group in the assessment of mathematics carried out after the intervention, since by improving the brain state of the student to attend to and process information better, they will have a better performance in a developing cognitive skill involved in mathematical thinking (Watson and Kelso, 2014; Cano-Estrada et al., 2022).

Several cognitive functions would benefit from the use of the Brain Gym in the experimental group of this research. For example, it is indicated that with the applied exercises levels of visual perception, concentration, hearing capacity, speed in problem solving, better brain state to generate neuronal interconnections and motor capacity would be improved. This cognitive difference would explain the better performance of the group that received the intervention with Brain Gym, which motivates us to continue carrying out new studies in favor of this technique that can benefit students in Latin America (Murtadho et al., 2019; Colina et al., 2021).

The limitations of the research were the difficulty of generating a random assignment of participants in each of the groups since the courses had been previously established, yet an attempt to go around this was to randomly select two groups.

In the future, and based on the results obtained in this research, it’s proposed to use Brain Gym at other levels of education. In hypothesis, in further research, the use of these types of approaches will bring benefits to complex and essential knowledge in Mathematics such as functions, algebra, geometry, measurement, statistics, and probabilities.

## Data availability statement

The raw data supporting the conclusions of this article will be made available by the authors, without undue reservation.

## Ethics statement

The studies involving human participants were reviewed and approved by Comité de Ética de la Universidad Indoamérica de Ecuador. Written informed consent to participate in this study was provided by the participants’ legal guardian/next of kin.

## Author contributions

CR-G: conceptualization, investigation, formal analysis, writing original draft, review and editing, and project administration. CA-V: investigation, writing original draft, and formal analysis. JC-C: investigation, formal analysis, and review and editing. All authors contributed to the article and approved the submitted version.

## Funding

This research was funded by Pontificia Universidad Católica del Ecuador and Universidad Tecnológica Indoamérica de Ecuador.

## Conflict of interest

The authors declare that the research was conducted in the absence of any commercial or financial relationship that could be construed as a potential conflict of interest.

## Publisher’s note

All claims expressed in this article are solely those of the authors and do not necessarily represent those of their affiliated organizations, or those of the publisher, the editors and the reviewers. Any product that may be evaluated in this article, or claim that may be made by its manufacturer, is not guaranteed or endorsed by the publisher.
